# Environmental DNA surveys detect distinct metazoan communities across abyssal plains and seamounts in the western Clarion Clipperton Zone

**DOI:** 10.1111/mec.15484

**Published:** 2020-06-14

**Authors:** Olivier Laroche, Oliver Kersten, Craig R. Smith, Erica Goetze

**Affiliations:** ^1^ Department of Oceanography School of Ocean and Earth Science and Technology University of Hawaii at Mānoa Honolulu HI USA; ^2^ Centre for Ecological and Evolutionary Synthesis University of Oslo Oslo Norway

**Keywords:** abyssal plains, CCZ, deep sea mining, meiofauna, metabarcoding, polymetallic nodules

## Abstract

The deep seafloor serves as a reservoir of biodiversity in the global ocean, with >80% of invertebrates at abyssal depths still undescribed. These diverse and remote deep‐sea communities are critically under‐sampled and increasingly threatened by anthropogenic impacts, including future polymetallic nodule mining. Using a multigene environmental DNA (eDNA) metabarcoding approach, we characterized metazoan communities sampled from sediments, polymetallic nodules and seawater in the western Clarion Clipperton Zone (CCZ) to test the hypotheses that deep seamounts (a) are species richness hotspots in the abyss, (b) have structurally distinct communities in comparison to other deep‐sea habitats, and (c) that seafloor particulate organic carbon (POC) flux and polymetallic nodule density are positively correlated with metazoan diversity. eDNA metabarcoding was effective at characterizing distinct biotas known to occur in association with different abyssal substrate types (e.g., nodule‐ and sediment‐specific fauna), with distinct community composition and few taxa shared across substrates. Seamount faunas had higher overall taxonomic richness, and different community composition and biogeography than adjacent abyssal plains, with seamount communities displaying less connectivity between regions than comparable assemblages on the abyssal plains. Across an estimated gradient of low to moderate POC flux, we find lowest taxon richness at the lowest POC flux, as well as an effect of nodule size on community composition. Our results suggest that while abyssal seamounts are important reservoirs of metazoan diversity in the CCZ, given limited taxonomic overlap between seamount and plains fauna, conservation of seamount assemblages will be insufficient to protect biodiversity and ecosystem function in regions targeted for mining.

## Abstract 2

see also the Perspective by Matthieu Leray and Ryuji J. Machida.

## 1 INTRODUCTION

The deep seafloor serves as a reservoir of biodiversity in the global ocean, with >80% of invertebrates at abyssal depths still undescribed (Smith, De Leo, Bernardino, Sweetman, & Martinez Arbizu, 2008; Snelgrove & Smith, 2002). The vast and remote abyssal plains remain largely unexplored (<0.01% sampled, Ramirez‐Llodra et al., 2010), although they represent the dominant topographical feature of the ocean seafloor (~70%). Abyssal plains experience high physical stability and are predominantly covered by fine sediments, providing habitat for diverse benthic communities (e.g., Glover & Smith, 2003; Hannides & Smith, 2003; Smith et al., 2008). This demersal fauna encounters a limiting allochthonous food supply and is characterized by slow growth, recruitment, reproduction and recovery rates following disturbance (Huvenne, Bett, Masson, Le Bas, & Wheeler, 2016; Ramirez‐Llodra et al., 2010).

Abyssal plains are punctuated by a multitude of seamounts (>1,000 m above bottom [mab]; Harris, Macmillan‐Lawler, Rupp, & Baker, 2014) that may serve as hotspots for biodiversity and potential refugia for populations impacted by environmental disturbances (Clark et al., 2010; Rowden, Schlacher, et al., 2010). Seamounts are subject to distinct hydrodynamic processes and physical conditions, including altered current velocity and organic matter deposition (Clark et al., 2010; White, Bashmachnikov, Arstegui, & Martins, 2008). They have also been shown in some cases to support higher abundance and biomass of benthic invertebrates than adjacent continental slopes (Beckmann & Mohn, 2002; Rogers, 1994; Rowden, Schlacher, et al., 2010), and to serve as stepping stones for dispersal (Cho & Shank, 2010; Leal & Bouchet, 1991; O’Hara, Consalvdey, Lavrado, & Stocks, 2010). Several emerging paradigms in seamount ecology have not been fully tested or contradictory evidence has been found, including the hypotheses that seamounts serve as species‐richness hotspots, and that they have distinct species composition or community structure, in comparison to adjacent deep seafloor habitats (McClain, Lundsten, Ream, Barry, & DeVogelaere, 2009; Rowden, Dower, Dower, Schlacher, Consalvey, & Clark, 2010). Seamounts have also been hypothesized to function as biogeographical “islands,” harbouring high levels of endemism (Koslow et al., 2001; McClain et al., 2009; Samadi et al., 2006; Stocks & Hart, 2007; Wilson & Kaufman, 1987), yet a number of studies have reported low levels of seamount endemism with greater sampling effort (Hall‐Spencer, Rogers, Davies, & Foggo, 2007; Samadi et al., 2006). Most seamounts studied to date have bathyal or shallower summit depths and occur in proximity to continental slopes; little is known about abyssal seamounts in remote areas of the central Pacific.

The Clarion Clipperton Zone (CCZ) deep seafloor holds significant metal and mineral resources in the form of polymetallic nodules (Ramirez‐Llodra et al., 2010). With dwindling onshore mineral reserves and security concerns over supply, there is renewed interest in mining the deep seafloor, as shown by a tripling in the number of exploration mining claims granted by the International Seabed Authority (ISA) in the past 8 years (Fukushima & Nishijima, 2017). The CCZ holds the highest abundance of polymetallic nodules of commercial interest of any region in the global ocean, with 16 of the 18 active nodule exploration contracts granted by the ISA within the CCZ (Wedding et al., 2015; Wedding et al., 2013; ISA website https://www.isa.org.jm). The ISA has designated nine no‐mining areas, termed Areas of Particular Environmental Interest (APEIs), each 160,000 km^2^, to safeguard regional biodiversity in the face of nodule mining (Wedding et al., 2013). The APEIs span large‐scale physical and biological gradients (Wedding et al., 2013, 2015), but there is limited ecological information available from APEIs, hindering accurate assessment of their regional representativity (De Smet et al., 2017; Gollner et al., 2017; Miller, Thompson, Johnston, & Santillo, 2019). Fundamental ecological knowledge, including levels of biodiversity, community composition, species ranges and population connectivity among habitats in these regions, remains largely unknown (Kaiser, Smith, & Arbizu, 2017).

Polymetallic nodules represent an important structuring element within the CCZ seafloor habitat, providing hard substrate microhabitats within the extensive soft sediments of the abyssal plains. Nodules support sessile organisms, such as xenophyophores, antipatharian corals and sponges, as well as numerous other megafaunal, meiofaunal and microbial taxa (Amon et al., 2016; Shulse, Maillot, Smith, & Church, 2017; Thiel, Schriever, Bussau, & Borowski, 1993; Vanreusel, Hilario, Ribeiro, Menot, & Arbizu, 2016; Veillette et al., 2007). Nodules influence the community composition and distribution of abyssal biota, and positively affect organismal abundance and diversity (e.g., Mullineaux, 1987; Shulse et al., 2017; Vanreusel et al., 2016; Veillette et al., 2007). Nodule mining will remove and bury the nodule, hard‐substrate habitats and cause resuspension of the upper ~5‐cm sediment layer (Oebius, Becker, Rolinski, & Jankowski, 2001; Thiel et al., 2001); thus, nodule mining is expected to have substantial disturbance effects on benthic communities (Glover & Smith, 2003; Jones, Amon, & Chapman, 2018). Seamounts within the CCZ might harbour refugial populations and provide larval sources for the hard‐substrate biota likely to be obliterated by large‐scale mining operations on the abyssal plains, but they remain almost entirely unstudied.

Environmental DNA (eDNA) metabarcoding surveys can provide baseline assessments of biodiversity that may circumvent some of the challenges of comprehensively sampling remote and highly diverse communities in deep ocean habitats (Boschen et al., 2016). Methods based on eDNA, herein defined to include both intra‐ and extracellular DNA, are particularly informative for detecting rare, cryptic and invasive species (Cristescu & Hebert, 2018; Kersten, Vetter, Jungbluth, Smith, & Goetze, 2019). Many species in the abyssal CCZ are undescribed (e.g., Amon et al., 2016; Tilot, Ormond, Moreno Navas, & Catalá, 2018), and whole community sequencing could provide a valuable baseline community assessment prior to mining, with limited dependence on taxonomic species descriptions. eDNA metabarcoding is increasingly being used to characterize and monitor marine ecosystems (Danovaro et al., 2016; Everett & Park, 2018; Goodwin et al., 2017), but has seen limited application in the deep sea. Recent eDNA studies on deep ocean sediments have shown high local heterogeneity, and a high proportion of uncharacterized species in eukaryotic communities (Dell’Anno, Carugati, Corinaldesi, Riccioni, & Danovaro, 2015; Guardiola et al., 2015, 2016; Lejzerowicz, Esling, & Pawlowski, 2014; Sinniger et al., 2016).

Using a multigene eDNA metabarcoding approach, we aimed to comprehensively characterize metazoan communities in three APEIs in the western CCZ (APEIs 1, 4 and 7), and test several hypotheses regarding diversity across environmental gradients in the abyssal benthos. First, we compare community composition and diversity between samples from three different substrates, seafloor sediments, polymetallic nodules and seawater from the benthic boundary layer (BBL), to evaluate how effectively eDNA metabarcoding distinguishes the distinct biotas known to occur in these different substrate types (e.g., Amon et al., 2016; Simon‐Lledó et al., 2019). We then test the hypotheses that deep seamounts (a) are species richness hotspots in the abyss, (b) have distinct community composition and biogeography in comparison to other deep sea habitats, and (c) that seafloor particulate organic carbon (POC) flux and polymetallic nodule density are positively correlated with metazoan diversity. We discuss our results in the context of future deep seabed mining and the potential importance of biodiversity hotspots to conservation of metazoan communities at the abyssal seafloor.

## 2 MATERIALS AND METHODS

### 2.1 Field sampling

**Figure 1:**
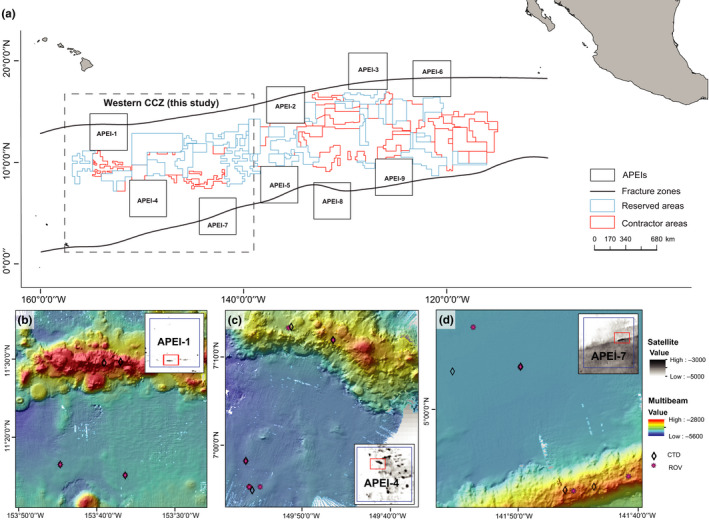
Maps of the study areas within the Clarion Clipperton Zone. (a) Overview of the CCZ and location of the APEIs. Sampling locations within APEI 1 (b), APEI 4 (c) and APEI 7 (d), with symbols for collection types and inset map of the seamount location within the APEI. APEI = Area of Particular Environmental Interest, designated as no‐mining areas by the ISA

Samples from seafloor sediment, polymetallic nodules and seawater were collected in APEIs 1, 4 and 7 within the western CCZ between May 22 and June 12, 2018 aboard cruise 18‐08 on the *RV Kilo Moana* (DeepCCZ cruise), using the *ROV Lu'ukai* (Figure 1). Sampling targeted one seamount and the adjacent abyssal plain habitat within each APEI. The sampled seamounts were elongate features with summit depths of 3,100 m (APEI 7), 3,500 m (APEI 4) and 3,900 m (APEI 1), all with summits >1,000 m above the surrounding abyssal plain. Adjacent abyssal plain sites were sampled >15 km away from the seamount ridgeline (APEI 7) or base (APEIs 4 and 1), with the expectation that this would be outside the “zone of influence” of the seamount, although limited data are available from the deep sea with which to estimate the appropriate scale of seamount influence. Large seamounts with shallow summit depths are relatively better studied, and for these features, seamount effects have been documented to a radius of up to 30 km. In the deep ocean, current velocities are generally an order of magnitude lower than in the energetic top 500 m of the water column; therefore, to be conservative, we chose a 15‐km buffer from the summit of the seamount to the nearest abyssal‐plain sampling sites. The seamount in APEI 1 was sampled for seawater only.

The *ROV Lu'ukai* was used to sample sediments and nodules, with three dives in APEI 7 (two abyssal plain, one seamount), three dives in APEI 4 (two abyssal plain, one seamount) and two abyssal plain dives in APEI 1, with two to five sediment cores collected for eDNA on each ROV dive. Seven‐centimetre‐diameter push cores were gently inserted vertically into the sediment by the ROV, sealed and recovered in the ROV work basket, and then horizontally sectioned onboard ship into 0–2 and 3–5‐cm sediment intervals for eDNA. Sterile syringes (60 ml; single‐use) were used to extract minicores from each sediment interval. Sediment processing gear and push‐core tubes were treated with 10% bleach and rinsed with double‐distilled water (ddH_2_O) between each ROV dive to prevent contamination. Slicing equipment was rinsed in ddH_2_O between cores. Two eDNA minicore technical replicates were taken for all cores from APEI 1. Samples were cryopreserved at −80°C until further processing. Polymetallic nodules were either collected in push cores, or by the manipulator arm of the ROV and placed in a sealed sample box (BioBox) for shipboard recovery. Once brought onboard ship, nodules were transferred to sterile whirl‐pack bags and cryopreserved (−80°C). Table S1 lists all ROV push cores sampled for eDNA.

Seawater samples were collected using conductivity–temperature–depth (CTD) casts with a rosette sampler with 24 × 10‐L Niskin bottles (SBE 911plus/917plus, SeaBird oxygen sensor [SBE43], Seapoint fluorometer, Wetlabs C‐Star transmissometer). A total of 12 CTD casts were conducted during the cruise, with two over the abyssal plain and two over the seamount within each APEI (Table S1). Niskin bottles were collected at seven depths within the water column: 5 mab, 50 mab, bathypelagic depths (3,000 m over plains, 2,500 or 2,000 m over seamounts), deep mesopelagic at 1,000 m, mesopelagic at 500 m, deep chlorophyll maximum (DCM; between 90 and 60 m), and 5 m in the near sea surface. Seawater volumes filtered were variable across depth, 5 L per replicate at 5 mab, 50 mab and bathypelagic depths, 4 L in the deep mesopelagic (1,000 m), 2 L in the mesopelagic (500 m), and 1 L at the DCM and in the near surface, with four to six replicates taken from each cast and depth. Field negative controls (ddH_2_O) were collected for each CTD cast, with filtration and handling as for all other bottles. Seawater was filtered onto 0.2‐µm sterile Supor filters (Pall) using 47‐mm inline polycarbonate filter holders and two peristaltic pumps. Filters were immediately preserved in 1 ml of RNALater (Invitrogen), flash frozen in liquid nitrogen, and held at −80°C until further processing. During the sampling process, carboys, tubing, plastics and the workspace were treated with 10% bleach for a minimum of 30 min to minimize cross‐contamination, followed by three ddH_2_O and three seawater rinses. To avoid contamination during sample collection, personal protective equipment included disposable laboratory coats and nitrile gloves for all involved personnel.

### 2.2 Sample processing and library preparation

eDNA was extracted from sediment samples using the PowerMax Soil kit (Qiagen) following the manufacturer's protocol. Approximately 10 g of homogenized sediment (mixed with a sterile metallic spatula) was used per extraction. Captured and purified DNA was eluted in 1 ml and then 4 ml ddH_2_O. Polymetallic nodules were weighed, and eDNA extraction was performed by first grinding and homogenizing nodules inside their whirl‐pack bag using a 16‐g ceramic pestle. Ten subsamples of ~500 mg per nodule were used for eDNA extraction with the FastDNA Spin kit according to the manufacturer's instructions. To obtain sufficient DNA for polymerase chain reaction (PCR) amplification, subsamples were pooled in pairs (mean DNA concentration of 0.382 ng/μl) and concentrated to ~1 ng/μl with the DNA Clean & Concentrator kit (Zymo Research), resulting in five replicates per nodule. eDNA from seawater samples was extracted with the DNeasy Plant Mini kit (Qiagen), using a modified protocol as described in Laroche, Kersten, Smith, and Goetze (2020). Due to low eDNA concentration in the 5‐ and 50‐mab samples, two replicates per collection point (2 × 5 L of filtered seawater for each depth) were pooled. For all sample types (seawater, sediment, nodules), an extraction blank was used to assess potential contamination during sample processing. All sample handling and DNA extraction steps were carried out in a dedicated laboratory free of PCR‐amplified DNA.

Eukaryotic communities were characterized by amplicon sequencing using two genetic markers, the V4 region of the 18S rRNA gene (~450 bp) and a fragment (~ 350 bp) of the mitochondrial cytochrome *c* oxidase subunit 1 (COI) gene. For 18S rRNA, the eukaryotic forward Uni18SF: 5′‐AGGGCAAKYCTGGTGCCAGC‐3′ and reverse primer Uni18SR: 5′‐GRCGGTATCTRATCGYCTT‐3′ primers (Zhan et al., 2013) were used. For COI, amplifications used the universal metazoan primers mlCOIintF: 5′‐GGWACWGGWTGAACWGTWTAYCCYCC‐3′ and jgHCO2198: 5′‐TAIACYTCIGGRTGICCRAARAAYCA‐3′ (Geller, Meyer, Parker, & Hawk, 2013; Leray et al., 2013). Details regarding library preparation can be found in Supplementary Material. Unprocessed sequencing reads are available from the NCBI Sequence Read Archive (SRA) under accession nos. SRR9199590 to SRR9199853.

### 2.3 Bioinformatic analysis

Samples were demultiplexed by their 8‐mer Nextera index, and then demultiplexed by target gene using cutadapt (version 1.8; Martin, 2011). Sample reads were denoised with the dada2 program (Callahan et al., 2016) implemented in qiime2 (version 2018.11; Boylen et al., 2018) using the default parameters. De novo chimera detection was performed using the consensus approach. Forward and reverse reads were truncated at 260 and 235 bp for 18S rRNA, and at 260 and 215 bp for COI, respectively, and merged using a perfect minimum overlap of 20 bp. Trimming of the 3′ end of the forward and reverse reads was performed to reduce Phred‐score‐based expected error of the sequences, and increase the yield of good quality, denoised reads. For 18S rRNA, taxonomic assignment for each read was performed with a naive Bayes classifier (Bokulich et al., 2018) implemented in qiime2 and trained on a trimmed SILVA 18S rRNA database (release 132 clustered at 99% similarity; Wang, Garrity, Tiedje, & Cole, 2007). For COI, taxonomic assignment was achieved using a combination of approaches that included the use of the classification trees (“insect”) classifier (version 5; Wilkinson, Davy, Bunce, & Stat, 2018), and megablast and blastn methods (Camacho et al., 2009) applied to the GenBank nucleotide (nt) database (Benson, Karsch‐mizrachi, Lipman, Ostell, & Wheeler, 2008). Complete description of the methods used in taxonomic assignment can be found in the Supporting Material.

### 2.4 Data analysis and statistics

Sequencing depth and recovered diversity per sample was investigated using rarefaction curves with the “vegan” r package (Oksanen et al., 2018). Prior to data analysis, sequences found in all negative controls, including field (ddH_2_O), DNA extraction and PCR blanks were investigated (Table S2) and removed from the data set. Sequences unidentified at the kingdom level or not part of Metazoa, and those originating from nonmarine taxa were also discarded (72% of 18S reads and 64% of COI reads). For COI, two data sets were explored, one using amplicon sequence variants (ASVs), and one of operational taxonomic units (OTUs) from ASVs clustered at 97% similarity using the default parameters of vsearch (Rognes, Flouri, Nichols, Quince, & Mahé, 2016) implemented in qiime2.2018‐11. The OTU‐level analysis aims to achieve putative species‐level taxonomic resolution. To remove pelagic legacy eDNA, or eDNA that derives from organisms living in overlying pelagic ecosystems, all ASVs found in the water column (from 5 m in the near sea surface to 2,000 m [seamount] or 3,000 m [abyssal plain]) were discarded from the deep‐sea samples (sediment, nodules, 5 mab and 50 mab BBL seawater samples), as in Laroche et al. (2020). To simplify analyses, sample data from both the 0–2‐ and 3–5‐cm sediment horizons were combined, representing eDNA collected from a total of 20 g of sediment per sample. Taxonomic composition of the sediment, polymetallic nodules and BBL samples was visualized with a cladogram containing a circular heatmap and barplots using graphlan (Asnicar, Weingart, Tickle, Huttenhower, & Segata, 2015) and the metacoder r package (Foster, Sharpton, & Grünwald, 2017). For this analysis, only taxa found in a minimum of five samples were included. ASV and OTU richness, estimated with the chao2 index, was used to compare alpha‐diversity between sample types, APEIs and habitats at base coverage. Base coverage is defined as the highest coverage value between minimum extrapolated values and maximum interpolated values (see Chao et al., 2014), and we use it as a metric for comparison among samples that standardizes for sampling coverage (or completeness). Calculations were performed using the inext r package (Hsieh, Ma, & Chao, 2016). Only sediment samples were considered for the comparison between APEIs and habitats (seamount, plain). ASVs and OTUs shared between sample types were investigated with Venn diagrams and the eulerr R package (Larsson, 2019). To avoid any bias from sampling coverage, data from each sample type was subsampled at equivalent coverage (determined by the chao2 index) with 50 iterations. Mean metazoan and phyla richness per sample and sample source were visualized with stacked barplots, plotted using the ggpubr R package (Kassambara, 2018).

Beta‐diversity analysis was conducted using unweighted UniFrac distance matrices (Lozupone & Knight, 2005) within phyloseq (McMurdie & Holmes, 2013), and visualized with nonmetric multidimensional scaling (nMDS) plots. The matrices were based on phylogenetic trees produced in qiime2 using the phylogeny align‐to‐tree‐mafft‐fasttree command (Katoh & Standley, 2013; Price, Dehal, & Arkin, 2010) and default parameters. The homogeneity of variance within sample type, APEI and habitat groups was analysed with the betadisper function of the vegan package. Differences in beta‐diversity between sample types, APEIs and habitats were assessed with pairwise permutational analysis of variance (PERMANOVA) in the vegan R package. The effect of nodule weight on community composition was assessed by PERMANOVA using the adonis function of the vegan package, with nodule weight nested within APEI. To correspond as closely as possible to traditional morpho‐taxonomy studies, both alpha‐ and beta‐diversity analyses were performed on the COI data clustered into OTUs at 97% similarity (putative species‐level differentiation).

Using presence/absence data, the proportion of taxa either unique to each habitat (seamounts, abyssal plains) and APEI, unique to a habitat but not to an APEI (“widespread habitat‐specific”) or found in diverse habitats and APEIs (“widespread nonspecific”) was visualized at both the ASV level (18S and COI) and OTU level (COI) using bar plots (plotted using the ggplot2 R package). For this analysis, only sediment samples were considered. To account for uneven sampling among APEI:Habitat combinations, the biogeography category assignment of each ASV/OTU was carried out by randomly subsampling each APEI:Habitat group to the sample size of the smallest group (e.g., two cores), and by performing 100 iterations. Differences in the proportion of unique, widespread habitat‐specific and widespread nonspecific taxa among habitats were tested with a Kruskal–Wallis rank sum test. The choice to use a nonparametric test was motivated by significant differences observed in group variance based on a Levene's test.

## 3 RESULTS

### 3.1 High‐throughput sequencing

A total of 10,315,003 and 17,202,778 reads were generated for 18S and COI, respectively (Table S3). Quality filtering, denoising, merging and chimera removal reduced 18S read counts by 54% and COI read counts by 40%, leaving an average of 20,040 and 43,572 good quality reads per sample for 18S and COI, respectively. ASVs found in sampling and extraction blanks were removed from all samples and are reported in Table S2. Rarefaction curves indicated that all but one sample (18S N‐26) were sufficiently sequenced to capture total amplicon within‐sample richness (reached an asymptote, Figures S1 and S2). This sample, along with two COI seawater samples with very few reads (<3,000 reads; W‐416‐417, W‐74‐75) were excluded from all downstream analyses.

While only 7% of 18S sequences could not be assigned to a domain, unclassified COI sequences at the level of domain represented 59% of reads. Once these unclassified reads were removed, the proportion of sequences derived from Metazoa was 30% for 18S and 90% for COI. Among sample types, seawater samples contained the lowest proportion of metazoan reads (20% [18S] and 78% [COI]). Protists (SAR supergroup) corresponded to 69% and 8% of all reads, respectively, while Fungi and Viridiplantae comprised less than 1% and 2% of 18S and COI reads. Keeping only metazoan taxa resulted in a total of 2,020 and 11,901 ASVs, and 1,308,427 and 2,802,156 reads for 18S and COI data, respectively. Removing ASVs found in the pelagic environment reduced the 18S data set to 1,759 ASVs and 839,626 reads, and the COI data set to 9,574 ASVs and 2,333,545 reads. Clustering COI ASVs at 97% similarity resulted in a total of 6,282 OTUs sampled in the abyss (all sample types).

### 3.2 eDNA taxonomic resolution

The level of taxonomic identification achieved varied substantially between marker genes, with much higher proportions of 18S rRNA reads assigned taxonomy at phylum to species levels (Table 1). For 18S, the phyla with the highest taxonomic resolution (ASVs identified to species level) with a minimum of 10 ASVs were Xenacoelomorpha (80%), Gastrotricha (73%), Chordata (50%) and Bryozoa (50%) (Table S4). Phyla with the lowest resolution included Nematoda (9% of ASVs identified to species), Ctenophora (9%), Nemertea (0%) and Loricifera (0%; Table S4). For COI data, the only phylum with high taxonomic resolution was Chordata, with 90% of OTUs identified at the species level (Table S4). Among the remaining most read‐count dominant phyla, the percentage of OTUs identified at species and genus levels (COI), respectively, were 5% and 23% for Echinodermata, 6% and 10% for Mollusca, 3% and 4% for Porifera, 2% and 6% for Annelida, 2% for Cnidaria and Arthropoda, and 0% for Platyhelminthes and Nemertea (Table S4).

**Table 1:** Mean percentage of metazoan amplicon sequence variants (ASVs; 18S) and operational taxonomic units (OTUs; COI) that could be assigned taxonomy at each level

Target gene	Phylum	Class	Order	Family	Genus	Species
18S rRNA	86.7	77.43	69.19	24.96	19.56	17.79
COI	18.59	7.68	3.21	1.48	0.88	0.69

### 3.3 Taxonomic composition and community diversity

#### 3.3.1 Sample type

**Figure 2:**
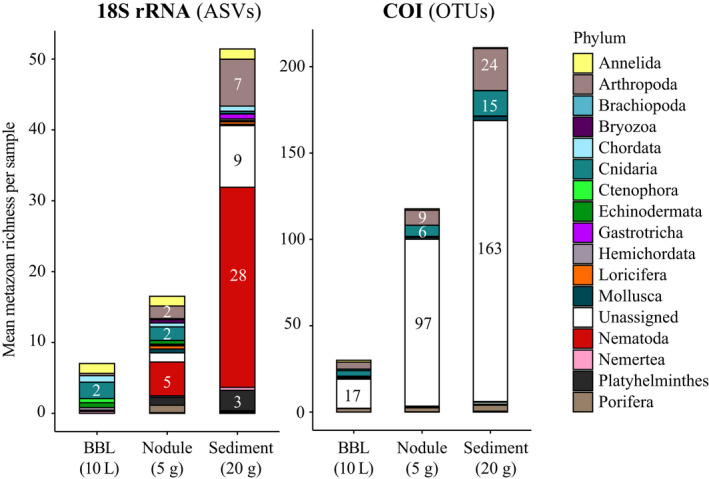
Barplots of mean metazoan 18S amplicon sequence variants (ASVs) and COI operational taxonomic units (OTUs), shown per sample type and coloured by phylum. BBL = benthic boundary layer. Numbers inside the histogram bars correspond to mean number of ASVs per phylum. Only the 10 most abundant phyla for 18S and COI are shown

**Figure 3:**
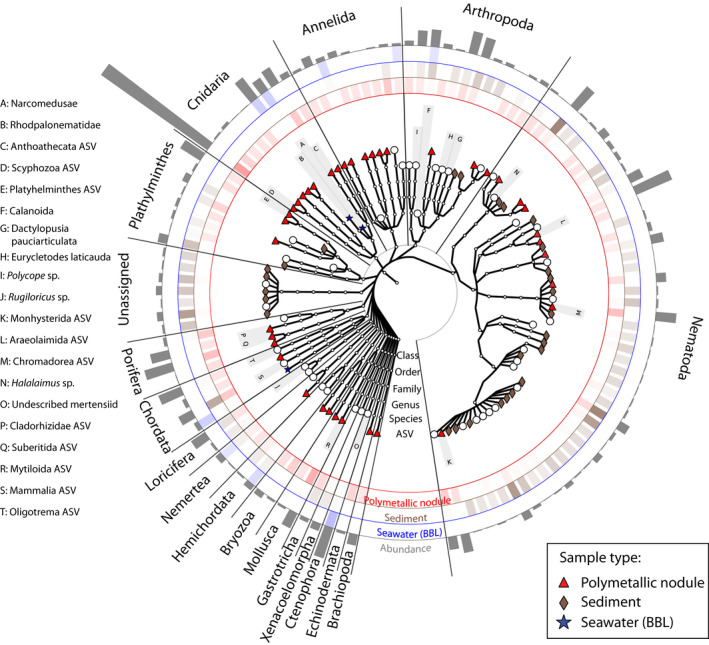
Cladogram with circular heatmap and barplots for the metazoan community resolved by 18S rRNA. The colour intensity in the circular heatmap corresponds to mean relative abundance in each sample type across the whole data set. The bar heights on the outside of the circle are proportional to the mean relative abundance of each taxon within the entire data set. Taxa found exclusively in one sample type are marked by a corresponding symbol: red triangle for nodules, grey diamond for sediment and blue star for BBL. Those found in more than one sample type are marked by a white circle. The 20 most abundant taxa at the tip of each branch are labelled with letters, and identified to highest taxonomic resolution (key at left). Only taxa found in a minimum of five samples were included. BBL = benthic boundary layer

Overall, a mean of seven, 17 and 51 unique 18S ASVs could be recovered per BBL seawater (10 L), polymetallic nodule (5 g) and sediment (20 g) sample (Figure 2). Metazoan diversity resolved in the 18S rRNA data was composed of 19 phyla, 35 classes, 71 orders and 97 families, largely dominated by nematodes (23% ASVs), cnidarians (16% ASVs), annelids (11% ASVs) and arthropods (10% ASVs). In terms of reads (Figure 3), nematode and arthropod (harpacticoid copepod) reads were predominantly found in both sediments and on nodules, while annelid, cnidarian, bryozoan, brachiopod, echinoderm, mollusc and poriferan reads were mostly present on nodules. Reads sampled in seawater mostly derived from cnidarians (Narcomedusae, Trachymedusae), ctenophores, and arthropods (calanoids) (Figure 3). Several taxa were found to be exclusive to a particular substrate type. Considering taxa present in at least five samples, 79 ASVs were found to be exclusively present on nodules (Table S5F), including brachiopods (Terebratulida), ascidians (Styelidae), corals (Isididae), bivalves (Veneroida, Mytiloida), hydroids (Ptilocodiidae), bryozoans, sponges (Cladorhizidae, Suberitida), turbellarian worms, polychaetes (Phyllodocidae, Syllidae) and scyphozoan cnidarians. Most of the 197 ASVs exclusive to sediments (five or more samples) were nematodes (Xyalidae, Comesomatidae, Enoplida), although 29 ASVs were classified as harpacticoids or arthropods and 14 were catenulid flatworms. A range of hydrozoan cnidarian groups as well as several other taxa were found to be exclusive to the BBL (e.g., Narcomedusae, Rhopalonematidae), but had lower recurrence across samples (occurrence in fewer than five samples; Table S5).

For COI, the mean number of recovered COI OTUs per sample was 30, 118 and 211 for BBL seawater (10 L), nodules (5 g) and sediments (20 g) (Figure 2). Overall, 19 phyla, 29 classes, 51 orders, and 55 families could be identified. Most of the OTU richness could be taxonomically assigned only to Metazoa (79% OTUs), with the remainder mostly assigned to arthropods (8% OTUs), cnidarians (6% OTUs), poriferans (3% OTUs), annelids and molluscs (1% OTUs). Although large numbers of reads and COI OTUs could not be taxonomically classified beyond Metazoa, their association to sample type and distribution across habitats could be resolved within the scope of our data. Of the ~30% of reads that could be assigned taxonomy to phylum or below, most cnidarian, annelid and echinoderm reads were sampled on nodules, sediments contained arthropods and cnidarians, and reads in BBL seawater samples were dominated by arthropods, cnidarians, poriferans, echinoderms and chordates (Figure S3). The proportion of unclassified metazoan ASVs was highest within nodule samples (82%), followed by sediment (77%) and BBL (56%) samples. Taxa that were restricted to a particular substrate type and present in at least five samples included sponges, such as hexactinellids and suberitids, for nodules (25 OTUs), and Chromadorea (nematodes) for sediments (four OTUs). Cetacea, Scombriformes and hydrozoan siphonophores, including Apolemiidae, Diphyidae, Forskaliidae and Sphaeronectidae, were found exclusively in BBL seawater, but had lower recurrence across samples in some cases (fewer than five samples; Figure S3, Table S6).

**Figure 4:**
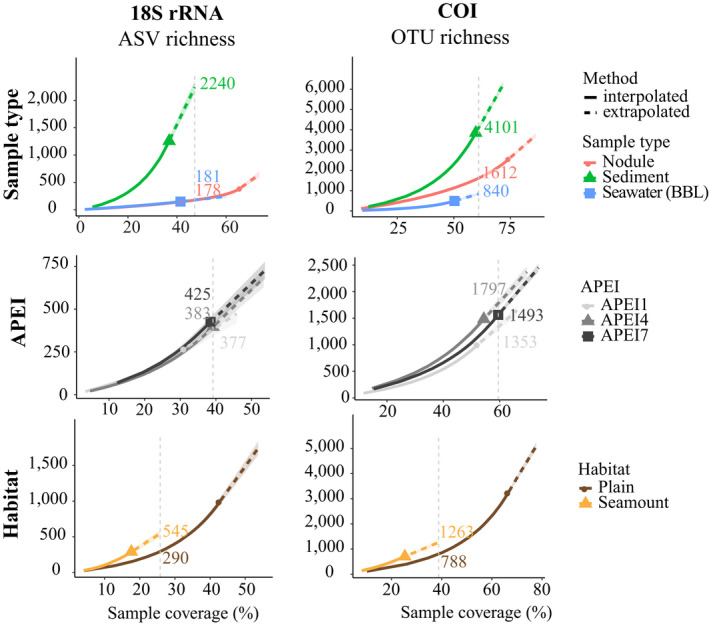
Metazoan 18S amplicon sequence variant (ASVs) and COI operational taxonomic unit (OTU) gamma diversity per APEI and habitat variable at base sampling coverage. ASV and OTU richness were estimated using chao2. Shaded coloured areas indicate the 95% confidence intervals obtained using a bootstrap method with 200 replicates. Coloured numbers in the plots represent number of ASVs/OTUs at base coverage. BBL = benthic boundary layer. For APEI and habitat comparisons, only sediment samples were included. Additionally, for the APEI comparison, seamount samples were excluded, as not all APEIs had seamount sediment data

Despite relatively low sampling coverage of ASV and OTU richness in sediment (37% and 60%, respectively) and seawater samples (41% and 50%, respectively), Figure 4 shows that at base coverage, or the highest coverage value between minimum extrapolated values and maximum interpolated values (Chao et al., 2014), sediments contained from 2.5 (COI) to 12.6 (18S) times the richness of BBL seawater or nodules. A significant difference can also be observed between seawater and nodules, but for COI data only, the latter containing twice as many estimated OTUs as seawater (Figure 4).

**Figure 5:**
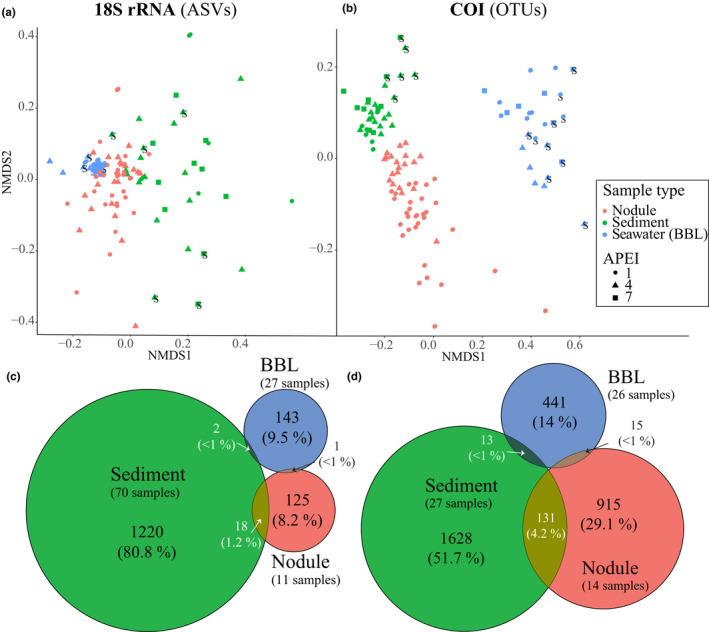
Community similarity across sample/substrate type and habitat. (a, b) Nonmetric multidimensional scaling plots (nMDS) of metazoan community dissimilarity, and (c, d) Venn diagrams illustrating shared metazoan amplicon sequence variants (ASVs; 18S) and operational taxonomic units (OTUs; COI) between sample types. nMDS plots are based on dissimilarity matrices using unweighted unifrac distance. Seamount samples in (a) and (b) are indicated by the letter “S.” Results in (c) and (d) represent mean values of 50 subsampling iterations. Subsampling was performed to normalize the number of samples per sample type at equivalent sampling coverage (coverage of 40% and 50% for 18S and COI, respectively), estimated using the chao2 index. BBL = benthic boundary layer, APEI = areas of particular environmental interest

Community composition differed significantly between sample types for both target genes, with stronger grouping by sample type within the COI data (Figure 5a,b). Pairwise PERMANOVA showed strongest dissimilarity between water samples and sediment or nodule samples for both target genes (Table S7). The analysis of homogeneity of variance among sample types was also significant (*p* < .043 both markers; Table S8), possibly due to the effect of habitat (seamount, plain).

Using a normalized approach in which the numbers and proportions of shared ASVs (18S) and OTUs (COI) between sample types were analysed at equivalent sampling coverage (40% and 50% for 18S and COI, respectively), in order to control for sampling effort, our analyses showed very little sequence overlap among substrates (Figure 5c,d). The highest proportion of shared sequences was found between sediment and nodules (mean of 1.2 and 4.2% of all ASVs and OTUs at equivalent coverage for 18S and COI, respectively; Figure 5c,d). Less than 1% of BBL ASVs and OTUs were found within sediment and nodule samples (Figure 5c,d).

#### 3.3.2 APEI

At the same sampling coverage, taxon richness tended to be slightly higher within APEI4 than APEI7, and lowest in APEI1 (Figure 4). Community composition was significantly different between APEIs for all sample types and target genes except 18S BBL seawater samples (*p* < .01; Table S9). Additionally, individual nodule weight significantly affected community composition, and to a greater extent than APEIs (*p* = .001; Table S9). Differences in community composition between APEIs were more pronounced in the COI data, where pairwise analysis found significant differences between all APEI combinations and for each sample type (*p* < .02; Table S10). In contrast, significant differences in community composition between APEIs in the 18S data were found only for nodules (*R* ^2^ = .088, *p* = .001, Table S10). Overall, the level of community dissimilarity between the different APEI pairwise comparisons were relatively similar (*R* ^2^ from .04 to .11; Table S10), with no clear association with geographical distance. A Mantel test using spatial coordinates and biological community dissimilarity matrices found significant correlations for nodules (*p* < .001, Table S11) and for COI sediment samples (*p* = .002, Table S11), but confirmed the absence of a spatial effect on BBL seawater and sediment samples for 18S data. Analysis of homogeneity of variance between APEIs found a significant difference between groups for the 18S data only (*p* = .044; Table S8).

#### 3.3.3 Habitat

**Figure 6:**
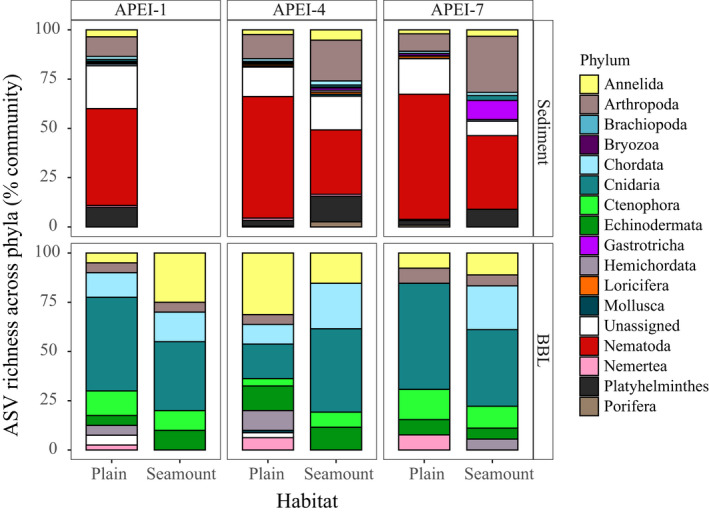
Community composition on seamounts and abyssal plains for each APEI. Relative 18S amplicon sequence variant (ASV) richness across phyla per sample, per habitat and per APEI. BBL = benthic boundary layer, indicating seawater sampled within the BBL; APEI = areas of particular environmental interest

The total sediment ASV and OTU gamma diversity was significantly higher (~2‐fold higher) on abyssal seamounts than on abyssal plains for both markers, as indicated by the absence of overlap in the confidence intervals in Figure 4. When analysed per APEI, only the APEI4 seamount had a significantly higher richness than the adjacent plain (Figure S4). Community composition was significantly different between habitats for both sediment (*p* ≤ .05; Table S9) and BBL seawater samples (*p* ≤ .024; Table S9), with no significant difference in group dispersion among habitats (Table S8). Relative diversity of arthropods and platyhelminths was higher in seamount sediments in comparison to adjacent abyssal plains (Figure 6), with a higher fraction of ASV diversity in nematodes in abyssal plain habitats. Comparison of BBL seawater between plains and seamounts found higher relative diversity of nemerteans on the plains and higher chordate diversity over seamount summits. Three families occurring in at least five samples were found to be specifically associated with abyssal plains: These included Nerillidae (annelid), and the nematode families Monhysteridae and Comesomatidae (Table S12).

### 3.4 Biogeography and range distributions across APEIs and habitats

**Figure 7:**
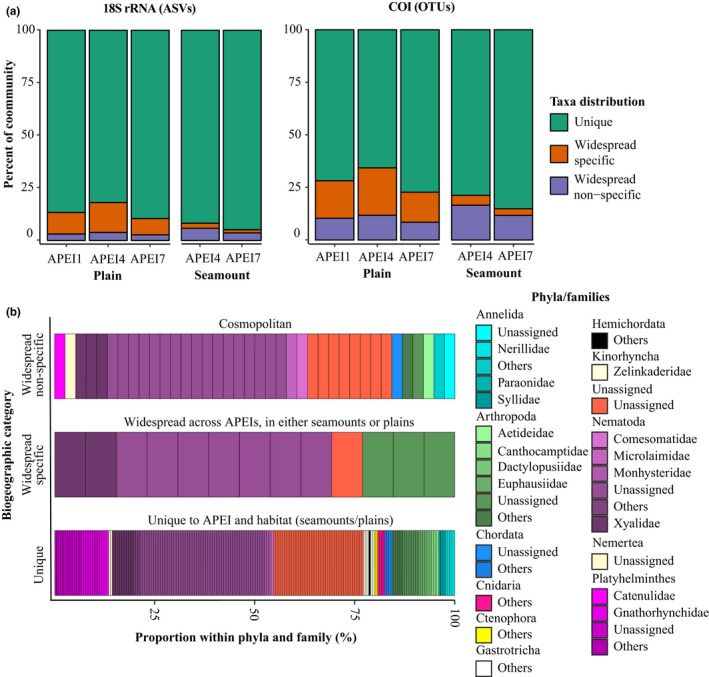
Community biogeography of abyssal seamounts and plains. (a) Proportion of sediment 18S amplicon sequence variants (ASVs) and COI operational taxonomic units (OTUs) found to be either unique to each APEI and habitat combination (Unique), found within more than one APEI but only one habitat (widespread‐specific), or found within more than one APEI and habitat (widespread nonspecific). (b) Taxonomic information for 18S ASVs within each biogeographical category. ASVs in (b) are delimited by thin black lines. In (b), results from all iterations were used to assign ASVs to taxonomic groups

The proportion of taxa unique to each APEI and bathymetric habitat was similar between 18S and COI data (Figure 7), and significantly higher for seamounts (mean of 90% and 82% for 18S and COI, respectively) than abyssal plains (mean of 85% and 72% for 18S and COI, respectively) (Kruskal–Wallis, *p* < .001, both markers; Table S13). The proportion of bathymetric habitat‐specific taxa, or those restricted to either seamounts or abyssal plains but found in different APEIs (widespread‐specific), was significantly lower for seamounts than for abyssal plains (Kruskal–Wallis, *p* < .001, Table S13). Conversely, taxa not specific to any habitat or APEI (cosmopolitan taxa) represented a slightly larger proportion of the community at seamount summits (8.5% and 14.5% for 18S and COI, respectively) than on the abyssal plains (5.9% and 11.2% for 18S and COI, respectively; Figure 7). Figure 7(b) shows that taxa found to be widespread across APEIs but bathymetrically restricted were exclusively arthropods, nematodes or unidentified metazoans. Cosmopolitan taxa included these groups as well as annelids, chordates, nemerteans and flatworms. Taxa unique to a habitat–APEI combination included the widest range of taxonomic groups, with cnidarians, ctenophores, gastrotrichs, hemichordates and kinorhynchs in addition to the more widespread groups (Figure 7b). In total, 26% of COI OTUs (56 of 212) that were found to be cosmopolitan in habitat association had ASVs, or COI haplotypes, that were specific to either seamount or abyssal plain habitats (for COI OTUs and ASVs observed in a minimum of five and three samples, respectively; >50 reads). This result suggests that approximately a quarter of cosmopolitan taxa may have population genetic structure, with COI haplotypes that are restricted in distribution to part of the species geographical range.

## 4 DISCUSSION

Deep‐sea ecosystems are under increasing anthropogenic pressure, with deep seabed mining a near‐term threat (Fukushima & Nishijima, 2017). Yet accurately characterizing biodiversity in the deep‐sea benthos using conventional surveys (e.g., visual, morphotaxonomy) requires extensive resources (Brandt et al., 2014), due to the remoteness of the habitat, challenging environmental conditions and relatively high numbers of rare invertebrate taxa. In this study, we attempt to address these issues by applying eDNA metabarcoding to assess metazoan diversity across substrates, habitats and large‐scale environmental gradients in the abyssal western CCZ.

Our results confirm that eDNA methods capture distinct communities as are known to occur in association with different substrates in the abyssal ocean (e.g., Amon et al., 2016; De Smet et al., 2017). This observation is important because one requirement for successful application of eDNA metabarcoding as a biomonitoring tool in the CCZ is that the method be sensitive enough to detect distinct communities that occur in close geographical proximity. We observed very distinct communities sampled in sediments, on polymetallic nodules and in the BBL seawater (Figure 5), with little organismal overlap (<5%) among ASVs (18S) and OTUs (COI) sampled at equivalent sampling coverage in distinct sample types (substrates). Taxa found exclusively on nodules were mostly sessile suspension feeders, including bryozoans, alcyonacean corals (Isididae), ascidians (Styelidae), brachiopods (Terebratulida), a number of sponge taxa (within Cladorhizidae, Hexactinellida and Suberitida), and bivalves (Venerida, Mytilida), among others (Tables S5 and S6), and this organismal list is broadly similar to nodule‐attached metazoans reported in previous work (Mullineaux et al. 1987, Amon et al., 2016; Vanreusel et al., 2016; Veillette et al., 2007). Taxa simultaneously found in association with both sediments and nodules were predominantly mobile organisms, including nematodes, arthropods and annelids, with the exception of a few sessile families, such as Arcidae (bivalve), Cladorhizidae (sponge) and Hexacrobylidae (ascidian). Organisms sampled exclusively in sediments were overwhelmingly nematodes (79 18S ASVs of 197 total ASVs that were exclusive to sediments), the dominant meiofaunal phylum. Although we expected that BBL plankton eDNA might settle to the seafloor, very few BBL ASVs and OTUs were observed in sediments (<6%) or nodules (≤2%).

We find evidence that abyssal seamounts may represent biodiversity hotspots for benthic organisms (e.g., 1.4–2.4 times higher richness, APEI 4), with distinct community composition and community biogeography in comparison to the adjacent abyssal plains in the western CCZ. Seamounts have long been hypothesized to be species richness hotspots (e.g., McClain, 2007), but evidence to support this hypothesis has been mixed (Rowden, Schlacher, et al., 2010), with several studies finding equivalent or lower richness on seamounts than on slopes or adjacent nonseamount areas (e.g., fishes, megafauna; Tracey, Bull, Clark, & MaCkay, 2004, O’Hara, 2007, Howell, Mowles, & Foggo, 2010). Results from this study provide new insights into the potential role of seamounts as biodiversity hotspots in that (a) our observations derive from seamounts that are more remote and with abyssal summit depths (~3,100, 3,500 m) that are deeper than the vast majority of seamounts studied to date, and (b) we use eDNA metabarcoding to estimate ASV/OTU richness, yielding greater taxonomic coverage and greater emphasis on smaller, more cryptic organisms than studies using conventional survey techniques. Our genetic eDNA data also have the asset that our observations are not limited by the current state of taxonomic knowledge for the assemblage. Given that > 80% of macrofaunal and meiofaunal invertebrates at abyssal depths are undescribed (George et al., 2014; Snelgrove & Smith, 2002), this is a considerable strength over morphology‐based measures. A number of mechanisms could cause elevated richness on seamounts, including higher habitat heterogeneity and/or heightened beta diversity reflecting faunal turnover across depth along the seamount flank, increased trophic input that supports elevated invertebrate abundance, biomass and diversity, or increased speciation rates due to the geographical isolation of seamounts (among others; McClain, 2007, Zeppilli, Bongiorni, Santos, & Vanreusel, 2014). The few previous quantitative studies of meiofaunal assemblages on seamounts have found that although summits may not have elevated richness relative to flanks or adjacent abyssal plain areas, they do have a very distinct nematode/copepod assemblage, with many species that are bathymetrically restricted in range and with high faunal turnover across depth and substrate on the seamount flank (enhancing beta diversity; George, 2013; George, Pointner, & Packmor, 2018; Zeppilli, Bongiorni, Cattaneo, Danovaro, & Santos, 2013; Zeppilli et al., 2014). Our results regarding distinct sediment community composition on seamount summits (Figure 6), largely driven by meiofaunal taxa, are broadly congruent with these previous observations. In the case of eDNA, one additional possible mechanism driving higher richness on seamounts is that seamount eDNA samples may integrate a larger spatial area than those on the plains, with bedload transport importing particulate matter and eDNA from microhabitat patches elsewhere on the seamount (beta diversity). Seamount summits are physically more open systems than abyssal plains, often with higher turbulence and current velocities (White et al., 2008), and eDNA may be transported into a site from nearby habitat patches. In this study, inference of the true richness on seamounts was constrained by the limited sampling coverage achieved (<30%). Further research is needed to confirm the hypothesis that seamounts are biodiversity hotspots across the abyss.

Seamounts have historically been perceived as isolated habitats, possibly harbouring high levels of endemism, due to their geographical isolation and hydrographic peculiarities (e.g., Taylor column formation), which can hinder larval dispersal and limit connectivity among populations (Clark et al., 2010; McClain et al., 2009; Samadi et al., 2006). Although limited evidence has been found supporting the seamount endemicity hypothesis (McClain et al., 2009; Rowden, Dower, et al., 2010), our results suggest that abyssal seamount benthic communities display less connectivity between APEIs than comparable communities on the abyssal plain. Specifically, a smaller proportion of the seamount community comprises taxa that are bathymetrically restricted but widespread across APEIs (seamount‐associated) than is observed for abyssal plain assemblages (plains‐associated). In other words, most seamount taxa with broad biogeographical ranges were not specific to a particular bathymetric habitat (seamounts, plains). In direct contrast, the majority of widespread (observed across different APEIs) abyssal plain taxa were not observed on seamounts and therefore may lack the capacity to colonize them. We also observe that a higher fraction of the seamount fauna is unique to habitat and APEI (endemics and pseudo‐endemics) than in abyssal plain habitats, at equivalent sampling coverage. In addition, several cosmopolitan OTUs were composed of sequence variants, or COI haplotypes, that were associated with a specific bathymetric habitat; this is initial tentative evidence of population genetic differentiation between plain and seamount populations within these putative species (26% of cosmopolitan taxa). Collectively, these observations support the inference that seamounts probably act both as biogeographical islands for taxa with limited dispersal ability, but also as stepping stones for dispersal for more cosmopolitan taxa (Miller & Gunasekera, 2017; Rowden, Dower, et al., 2010). Other studies report mixed support for seamounts as stepping stones for dispersal (e.g., O’Hara et al., 2010; Wilson & Kaufman, 1987), and taxon‐specific traits related to dispersal ability probably drive these broader biogeographical trends.

Abyssal ecosystems are strongly modulated by the flux of detrital material originating from the upper ocean due to food limitation in the abyss (Smith et al., 2008). Both the abundance and the diversity of macrofaunal invertebrates have been shown to positively correlate with POC flux (De Smet et al., 2017; Rex et al., 2006; Smith et al., 1997). Polymetallic nodules also enhance the abundance and regional diversity of the deep‐sea benthos as they provide hard substrate in an otherwise soft‐bottom environment for a range of sessile epifauna (Amon et al., 2016; Vanreusel et al., 2016; Veillette et al., 2007). APEIs sampled in this study span a range of moderate to low POC flux (Table 2; Table S14; Lutz, Caldeira, Dunbar, & Behrenfeld, 2007; Smith et al., 2019; Wedding et al., 2013) and high to low polymetallic nodule abundance (Table 2; ; Morgan et al., 2010; Smith et al., 2019). Overall, taxon richness was lowest within APEI 1, at lowest POC flux, and highest within APEI 4, at moderate POC flux and in a region containing both soft sediment habitat and high nodule abundance. Significant differences in sediment‐community composition were observed between APEIs. While spatial distance may be partly responsible for these differences, at least in the COI data, these results support the idea that POC flux and/or nodule density positively affect community diversity. We also find that nodule size, measured here as weight, influenced community composition. While this relationship was not observed in De Smet et al. (2017), it is concordant with results from Simon‐Lledó et al. (2019), suggesting nodule‐size preferences among taxa.

**Table 2:** Mean of estimated particulate organic carbon (POC) flux (gC m^−2^ yr^−1^) and polymetallic nodule abundance (kg m^−2^) at our sampling sites within each APEI

APEI	POC flux		Nodule abundance	
	Mean	*SD*	Mean	*SD*
1	1.13	0.025	2.14	0.311
4	1.40	0.041	5.83	0.089
7	1.88	0.060	0.58	0.002

Note

Estimates of POC flux derive from the global model reported in Lutz et al. (2007), and nodule abundance from the geological model described in ISA Technical Study No. 6 (also see Table S14).

Abbreviation: APEI, Area of Particular Environmental Interest.

eDNA metabarcoding could be a powerful and cost‐effective method of assessing biodiversity in baseline surveys of the deep sea. However, one of the primary limitations is the low representation of deep‐sea organisms in reference sequence databases (Kersten et al., 2019; Lacoursière‐Roussel et al., 2018; Wangensteen, Palacín, Guardiola, & Turon, 2018). In this study, only 25% and 1.5% of 18S and COI metazoan sequences could be assigned to family. This problem was especially pronounced in the COI data, where ~ 19% of metazoan reads could only be assigned to phylum. While many of the unassigned sequences probably derive from undescribed organisms that are new to science, a large fraction probably also corresponds to fully described taxa that lack representative DNA barcodes (see Lacoursière‐Roussel et al., 2018). The absence of taxonomic, and therefore ecological, information hinders our capacity to understand deep‐sea ecosystem processes and design and implement effective conservation measures. It is imperative that we continue allocating time and resources to describing new species, and augmenting reference databases with DNA barcodes for described species (Glover, Wiklund, Chen, & Dahlgren, 2018). Given our results, efforts should be directed towards the characterization of meiofaunal taxa in particular, as there is very high, but unclassified, diversity in sediments.

## 5 CONCLUSIONS AND MANAGEMENT IMPLICATIONS

Our results suggest that abyssal seamounts are important reservoirs of metazoan diversity in the abyssal CCZ, with elevated taxon richness relative to abyssal plains habitats. We observed distinct community composition on seamounts (as in Zeppilli et al., 2013; Zeppilli et al., 2014 and George et al., 2018), and limited taxonomic overlap with the adjacent abyssal plain assemblages (499 OTUs [16%] and 379 OTUs [19%] for APEIs 4 and 7, respectively), implying that even if seamount populations persist within claim areas during large‐scale seabed mining, they will not serve as major source populations to reseed disturbed areas of the adjacent abyssal plains. Conservation of these biologically distinct communities is important, but insufficient to ensure preservation of viable populations of the dominant abyssal plain fauna. We observed fairly large range distributions (up to 1,500 km) for 2.4% of the plains fauna (COI OTUs cosmopolitan across APEIs 1, 4 and 7 and present in at least five samples), suggesting that some species are distributed across spatial scales bridging APEIs and claim areas. The majority of OTUs/ASVs, however, were rare and limited to small spatial areas in our material, and so we cannot reject the hypothesis that they have restricted species ranges. In accordance with other studies, we also find highest metazoan richness in regions with both substantial nodule cover and soft sediment habitats, as well as moderate POC flux, environmental variables that have been shown to correlate with a higher abundance and diversity of megafaunal invertebrates within the CCZ (e.g., Amon et al., 2016; De Smet et al., 2017; Vanreusel et al., 2016). Finally, in this first eDNA study for the western CCZ, we demonstrate that eDNA metabarcoding could be a powerful survey tool for assessing community diversity in the context of seabed mining impacts. The taxonomic resolution is comparable to or higher than that typically obtained using image‐based survey techniques, and the communities detected are tightly linked to substrate type (nodules, sediments). Additional efforts to expand reference databases through DNA barcoding will enhance the classification power of eDNA methods, enabling more useful assessments and testing of long‐standing deep‐sea ecological hypotheses.

## AUTHOR CONTRIBUTIONS

E.G., O.K., O.L. and C.R.S. designed the study. E.G., O.K. and C.R.S. conducted fieldwork and sampling at sea. O.L. generated the data and performed analyses, and O.L. wrote the manuscript with intellectual contributions from all co‐authors. E.G. and C.R.S. provided grant and equipment support.

## Supporting information

Supplementary Material

Table S1

Table S2

Table S3

Table S4

Table S5

Table S6

Table S12

Table S14

## Data Availability

Unprocessed sequences are accessible from the NCBI Sequence Read Archive (SRA) under accession nos. SRR9199590 to SRR9199853. Metadata for the samples are available in the Supporting Information (Laroche, Oliver, Smith, & Goetze, 2019).
