# Supplementary material for: Environmental DNA surveys detect distinct metazoan communities across abyssal plains and seamounts in the western Clarion Clipperton Zone
Source: Mol Ecol. 2020 Jun 14;29(23):4588–604. doi: 10.1111/mec.15484 (PMC7754508; doi:10.1111/mec.15484)
Supplement: Supplementary file 1 — Supplementary Material [file MEC-29-4588-s001.docx]

**Supplemental Information for:**

**Environmental DNA surveys detect distinct metazoan communities across abyssal plains and seamounts in the western Clarion Clipperton Zone**

Olivier Laroche^1*^, Oliver Kersten^2^, Craig R. Smith^1^, Erica Goetze^1^

*^1^ Department of Oceanography, School of Ocean and Earth Science and Technology, University of Hawaii at Manoa, Honolulu, USA;*

*^2^ Centre for Ecological and Evolutionary Synthesis. University of Oslo, Norway;*

* Corresponding author,

Current address:

Institute of Marine Research,
PO Box 6606 Langnes
9296 Tromsø, Norway

E-mail: olli.laroche@gmail.com

**Table of Contents:**

| **PCR amplification and Library preparation** | Page 2 |
| --- | --- |
| **Bioinformatic analysis** | Page 3-4 |
| **Table S7** | Page 5 |
| **Table S8** | Page 5 |
| **Table S9** | Page 6 |
| **Table S10** | Page 6 |
| **Table S11** | Page 7 |
| **Table S13** | Page 7 |
| **Figure S1** | Page 8 |
| **Figure S2** | Page 9 |
| **Figure S3** | Page 10 |
| **Figure S4** | Page 11 |

**PCR amplification and Library Preparation**

Eukaryotic communities were characterized by amplicon sequencing using two genetic markers, the V4 region of the 18S rRNA gene (approximately 450 base pairs [bp]) and a fragment (ca. 350 bp) of the mitochondrial COI gene. For 18S rRNA, the eukaryotic forward Uni18SF: 5′-AGG GCA AKY CTG GTG CCA GC-3′ and reverse primers Uni18SR: 5′-GRC GGT ATC TRA TCG YCT T-3′ primers (Zhan et al., 2013) were used; For COI, the ﻿universal metazoan primers mlCOIintF: 5′-GGW ACW GGW TGA ACW GTW TAY CCY CC-3′ and jgHCO2198: 5′-TAI ACY TCI GGR TGI CCR AAR AAY CA-3′ (Leray et al., 2013) were used. In each case, primers were modified to include Illumina^TM^ overhang adaptors. The 18S rRNA PCR reactions consisted of 12.5 μL of MyFi™ Mix (Bioline), 0.5 μL of each primer (10 µM), 0.6 μL of bovine serum albumin (300 µM; BSA; Sigma), 1-3 μL of template DNA (min 2.5 ng per reaction) with ddH_2_O added to reach a total volume of 25 μL. The reaction cycling conditions were: 95°C for 3 min, followed by 35 cycles of 95°C for 15 s, 55°C for 30 s, 72°C for 15 s, with a final extension step at 72°C for 1 min. For COI, the PCR reaction was similar except that primers and BSA were added at 1 μL each. Cycling conditions for COI were: 95°C for 3 min, followed by 45 cycles of 95°C for 15 s, 50°C for 30 s, 72°C for 15 s, with a final extension step at 72°C for 1 min. Each PCR experiment included a negative control (no template DNA). PCRs were performed in duplicate and pooled in order to reduce the effect of stochasticity in amplification. Amplicons were purified with AMPure® XP beads (Beckman Coulter®, Indiana, USA) and quantified using a Qubit® Fluorometer (Life Technologies) following the manufacturer’s instructions. DNA concentration was normalised to 2 ng/μL with ddH_2_O, and 18S rRNA and COI amplicons from same sample were pooled together on a ratio of 1.25 ng of 18S rRNA / 1 ng of COI to account for the larger fragment size of the former. Samples, including two negative controls, were given to the Advanced Studies in Genomics Proteomics and Bioinformatics (ASGPB) of the University of Hawaii at Manoa (HI, USA) for indexing with the Nextera^TM^ DNA library Prep Kit (Illumina, California, USA). Samples were pooled into two libraries and sequenced on two MiSeq Illumina^TM^ runs using V3 chemistry and paired-end sequencing (2×300 bp). Each library contained a negative control to assess potential contamination during the second PCR step and sequencing.

**Bioinformatic analysis**

For 18S rRNA, taxonomic assignment for each read was performed with a naive Bayes classifier (Bokulich et al., 2018) implemented in Qiime2, trained on a trimmed SILVA 18S rRNA database (release 132 clustered at 99 % similarity; Quast et al., 2013) using the Uni18SF and Uni18SR primer pair (perfect match required by the qiime feature-classifier extract-reads function). The 18S V4 region is highly conserved (Wu et al. 2015), and ‘species’ level assignments that derive from this classification procedure may include a number of close relatives that have 100% sequence identity and cannot be differentiated at this marker. For COI, taxonomic assignment was achieved using a combination of approaches to reduce the number of unassigned sequences and increase taxonomic resolution. Sequences were assigned taxonomy using three approaches: 1) the classification trees (‘insect’) classifier (version 5; Wilkinson et al., 2018) trained on the Midori database (Machida, Leray, Ho, & Knowlton, 2017) and marine sequences from the GenBank nucleotide (nt) database (Benson, Karsch-mizrachi, Lipman, Ostell, & Wheeler, 2008); 2) megablast from blastn application (options: -evalue 0.001 -max_target_seqs 5 -task **megablast** -perc_identity 0.8; Camacho et al., 2009); and 3) blastn (options: -evalue 0.001 -max_taget_seqs 5 -task **blastn)** on the entire GenBank nt database. For the blastn methods, taxonomy returned from each hit (max 5 per query sequence) was assigned to the lowest common ancestor among hits. To avoid over-classification, this assignment was then corrected based on a minimum percent identity value for each taxonomic rank. These values were 97, 95, 87, 83, 81, 79 and 71% at the species, genus, family, order, class, phylum and kingdom level respectively. For example, if a hit had 87% percent identity with a query sequence, the returned taxonomy included up to family level, discarding species and genus information. Calculation of the percent identity thresholds were determined by 1) trimming the Midori database (version 20180221), keeping only marine taxa captured by the COI primers used in this study, 2) performing local pairwise alignment (Smith-Waterman) and computing percent identity between all sequence pairs using the Biostrings R package (Pagès, Aboyoun, Gentleman, & DebRoy, 2019) and, 3) for each taxonomic rank, computing the mean percent identity and standard deviation between all pairs of sequences at the mean (see table below). The formula used to calculate percent identity was 100 * number of identical positions / (aligned positions plus internal gaps), as calculated by blast. As a conservative measure, the mean percent identity at each taxonomic rank was computed between taxa from different lower taxonomic groups. For example, the mean percent identity at family level was based on pairwise percent identity values between sequences originating from different genera. The percent identity thresholds were calculated by adding the standard deviation to the mean. Finally, results from the Insect classifier and the blastn approaches were collated, and in the absence of conflicting results among methods, taxonomy was retained from the method with the highest resolution. In case of conflict, the lowest common ancestor among the different approaches was assigned.

As a side note, we tried the RDP classifier with a database containing non-redundant metazoan sequences from the Midori (Machida et al., 2017) and Bold (Ratnasingham and Hebert, 2007) databases for COI data, but it performed poorly in terms of taxonomic resolution. When using curated databases, the RDP classifier was outperformed by the Insect R classifier, which is why we retained the latter. However, a very high number of unclassified sequences remained using curated databases, which is why we decided to use a combined approach with megablast and blastn as well as the Genbank database.

Mean percent identity and standard deviation per taxonomic rank, based on analysis of the MIDORI database (version 20180221).

| **Mean**  **percent identity** | **Standard**  **deviation** | **Taxonomic rank** |
| --- | --- | --- |
| 68.3 | 3.09 | Kingdom |
| 75.96 | 3 | Phylum |
| 78.48 | 2.81 | Class |
| 78.73 | 4.44 | Order |
| 82.33 | 4.91 | Family |
| 90.13 | 6.6 | Genus |

* Percent identity based on the number of identical positions / (aligned positions plus internal gaps) x 100. As a conservative measure, the mean percent identity at each taxonomic rank was computed between taxa from different lower taxonomic groups.

**Table S7.** Pairwise permutational analysis of variance in community composition based on unweighted unifrac dissimilarity matrices between sample types (999 permutations). BBL = benthic boundary layer.

| **Dataset** | **Target gene** | **Pair-wise**  **combination** | **R^2^** | **Adjusted p-value** |
| --- | --- | --- | --- | --- |
| All deep-sea  samples | 18S rRNA | Sediment vs BBL | 0.099 | **0.003** |
|  |  | Sediment vs Nodules | 0.050 | **0.003** |
|  |  | Nodules vs BBL | 0.080 | **0.003** |
|  | COI | Sediment vs BBL | 0.110 | **0.003** |
|  |  | Sediment vs Nodules | 0.053 | **0.003** |
|  |  | Nodules vs BBL | 0.122 | **0.003** |

**Table S8.** Multivariate analysis of homogeneity of group dispersions in community composition based on unweighted unifrac dissimilarity matrices between sample types, APEI and habitats (999 permutations). APEI = Area of particular environmental. Permutations = 999.

| **Dataset** | **Target gene** | **Factor** | **F value** | **p-value** |
| --- | --- | --- | --- | --- |
| All deep-sea  samples | 18S rRNA | Sample type | 3.242 | **0.043** |
|  |  | APEI | 3.222 | **0.044** |
|  |  | Habitat | 2.499 | 0.117 |
|  | COI | Sample type | 5.383 | **0.006** |
|  |  | APEI | 0.804 | 0.451 |
|  |  | Habitat | 0.042 | 0.838 |

**Table S9.** Permutational analysis of variance of community composition based on unweighted unifrac distance matrices per sample type between APEIs (999 permutations). For sediment and water samples, habitat was nested within APEI. Since nodules were collected over abyssal plains only, the nested factor for nodules included nodule weight only. BBL = benthic boundary layer.

| **Dataset** | **Target gene** | **Variables** | **R^2^** | **p-value** |
| --- | --- | --- | --- | --- |
| Sediment | 18S rRNA | APEI | 0.085 | **0.009** |
|  |  | APEI:Habitat | 0.078 | **0.054** |
|  | COI | APEI | 0.089 | **0.001** |
|  |  | APEI:Habitat | 0.107 | **0.001** |
| Nodules | 18S rRNA | APEI | 0.088 | **0.001** |
|  |  | APEI:Nodule weight | 0.136 | **0.001** |
|  | COI | APEI | 0.066 | **0.001** |
|  |  | APEI:Nodule weight | 0.081 | **0.001** |
|  | 18S rRNA | APEI | 0.086 | 0.144 |
| BBL |  | APEI:Habitat | 0.194 | **0.024** |
|  | COI | APEI | 0.137 | **0.001** |
|  |  | APEI:Habitat | 0.159 | **0.001** |

**Table S10.** Pairwise permutational analysis of variance in community composition based on unweighted unifrac dissimilarity distances per sample type between APEIs (999 permutations). BBL = benthic boundary layer.

| **Dataset** | **Target gene** | **Pairwise**  **combination** | **R^2^** | **Adjusted p-value** |
| --- | --- | --- | --- | --- |
| Sediment | 18S rRNA | APEI1 vs APEI4 | 0.069 | 0.054 |
|  |  | APE1vs APEI7 | 0.084 | 0.177 |
|  |  | APEI4 vs APEI7 | 0.049 | 0.195 |
|  | COI | APEI1 vs APEI4 | 0.061 | **0.015** |
|  |  | APE1 vs APEI7 | 0.100 | **0.003** |
|  |  | APEI4 vs APEI7 | 0.056 | **0.006** |
| Nodules | 18S rRNA | APEI1 vs APEI4 | 0.088 | **0.001** |
|  | COI | APEI1 vs APEI4 | 0.066 | **0.001** |
|  | 18S rRNA | APEI1 vs APEI4 | 0.065 | 0.636 |
| BBL |  | APE1 vs APEI7 | 0.042 | 1 |
|  |  | APEI4 vs APEI7 | 0.113 | 0.417 |
|  | COI | APEI1 vs APEI4 | 0.108 | **0.003** |
|  |  | APE1vs APEI7 | 0.101 | **0.003** |
|  |  | APEI4 vs APEI7 | 0.107 | **0.003** |

**Table S11.** Results of Mantel tests for correlation between geographic distance and biological community dissimilarity using 999 permutations and based on unweighted unifrac distance matrices. BBL = benthic boundary layer.

| **Dataset** | **Target gene** | **r** | **Simulated p-value** |
| --- | --- | --- | --- |
| Sediment | 18S rRNA | 0.119 | 0.124 |
|  | COI | 0.285 | **0.002** |
| Nodules | 18S rRNA | 0.195 | **<0.001** |
|  | COI | 0.314 | **<0.001** |
| BBL | 18S rRNA | 0.231 | 0.096 |
|  | COI | 0.212 | 0.088 |

**Table S13.** Kruskal-Wallis rank sum test of differences in the proportion of taxa found to be either unique to each area of particular interest (APEI) and habitat combination (Unique), found within more than 1 APEI but only one habitat (widespread-specific) or found within more than 1 APEI and habitat (widespread non-specific). ASV = amplicon sequence variant, OTU = operational taxonomic unit.

| **Dataset** | **Category** | **chi-squared** | **p-value** |
| --- | --- | --- | --- |
| 18S rRNA | Unique | 208.7 | **< 0.001** |
|  | Widespread specific | 276.06 | **< 0.001** |
|  | Widespread non-specific | 16.726 | **< 0.001** |
| COI (ASVs) | Unique | 205.5 | **< 0.001** |
|  | Widespread specific | 244.52 | **< 0.001** |
|  | Widespread non-specific | 51.796 | **< 0.001** |
| COI (OTUs) | Unique | 228.12 | **< 0.001** |
|  | Widespread specific | 275.32 | **< 0.001** |
|  | Widespread non-specific | 98.35 | **< 0.001** |


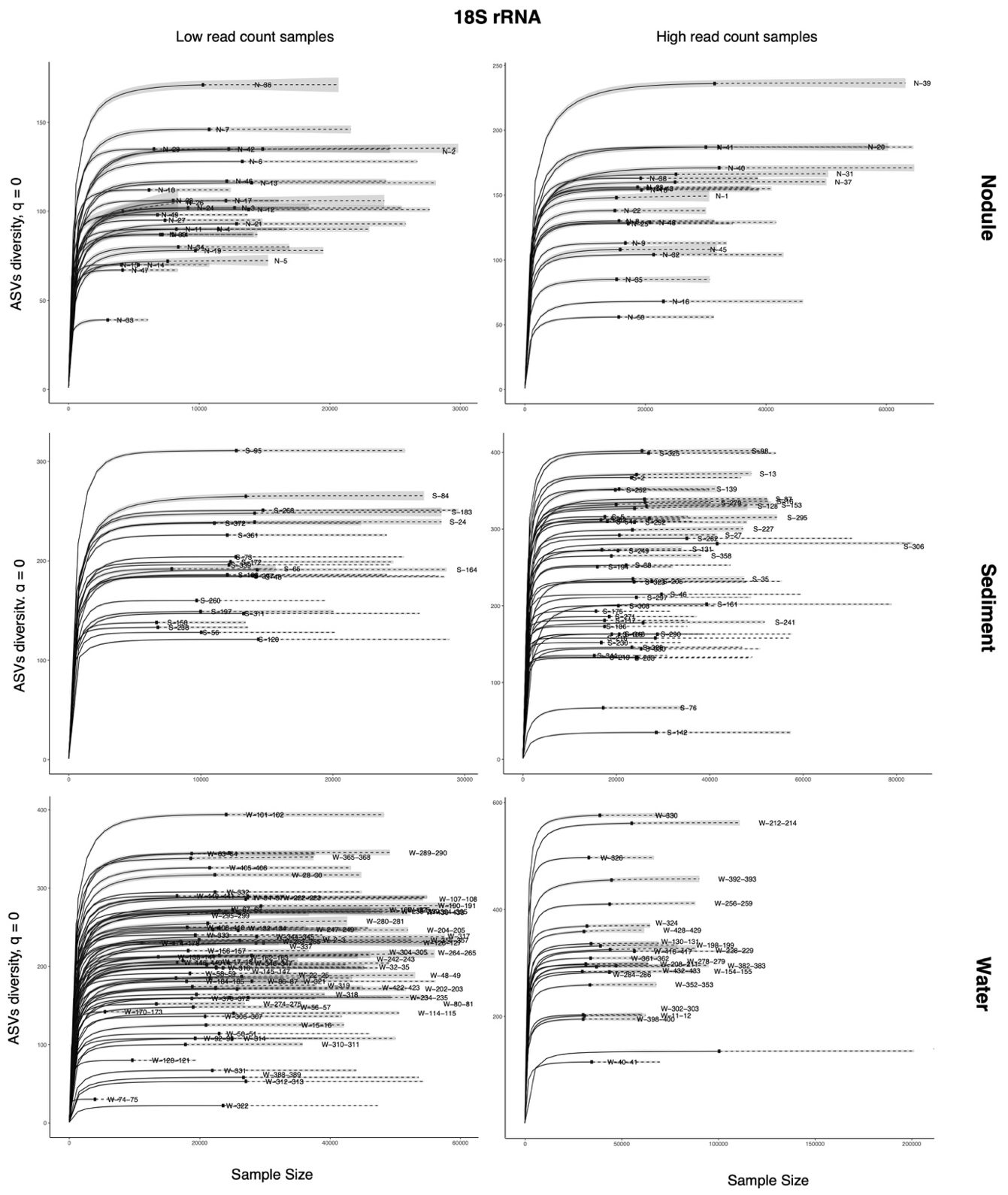


**Figure S1.** Rarefaction curves of 18S ASV richness across sequencing depth (Sample size) for all samples, plotted by sample type (polymetallic nodule, sediment, water). Sample names as labeled, with sampling metadata as reported in Table S1. Extrapolation values (dotted lines) are based on Chao1 estimator. Grey shaded areas correspond to the 95 % confidence interval. Samples included in the low read count section of the figure (left side) had read counts below 15,000 reads for nodule and sediment, and below 30,000 for water.


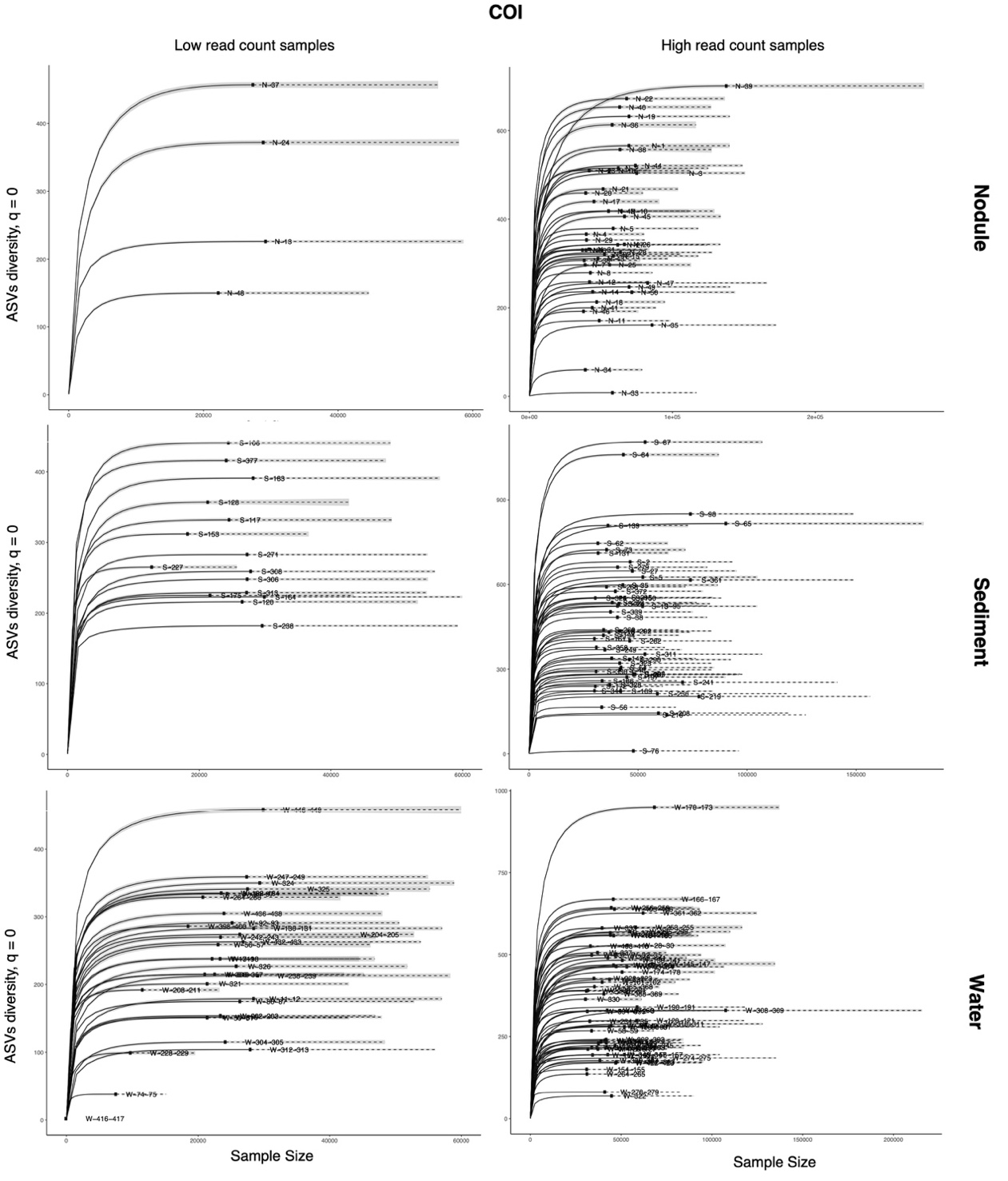


**Figure S2.** Rarefaction curves of COI ASV richness across sequencing depth (Sample size) for all samples, plotted by sample type (polymetallic nodule, sediment, water). Sample names as labeled, with sampling metadata as reported in Table S1. Extrapolation values (dotted lines) are based on Chao1 estimator. Grey shaded areas correspond to the 95 % confidence interval. Samples included in the low read count section of the figure (left side) had read counts below 30,000 reads for nodule, sediment, and water samples.


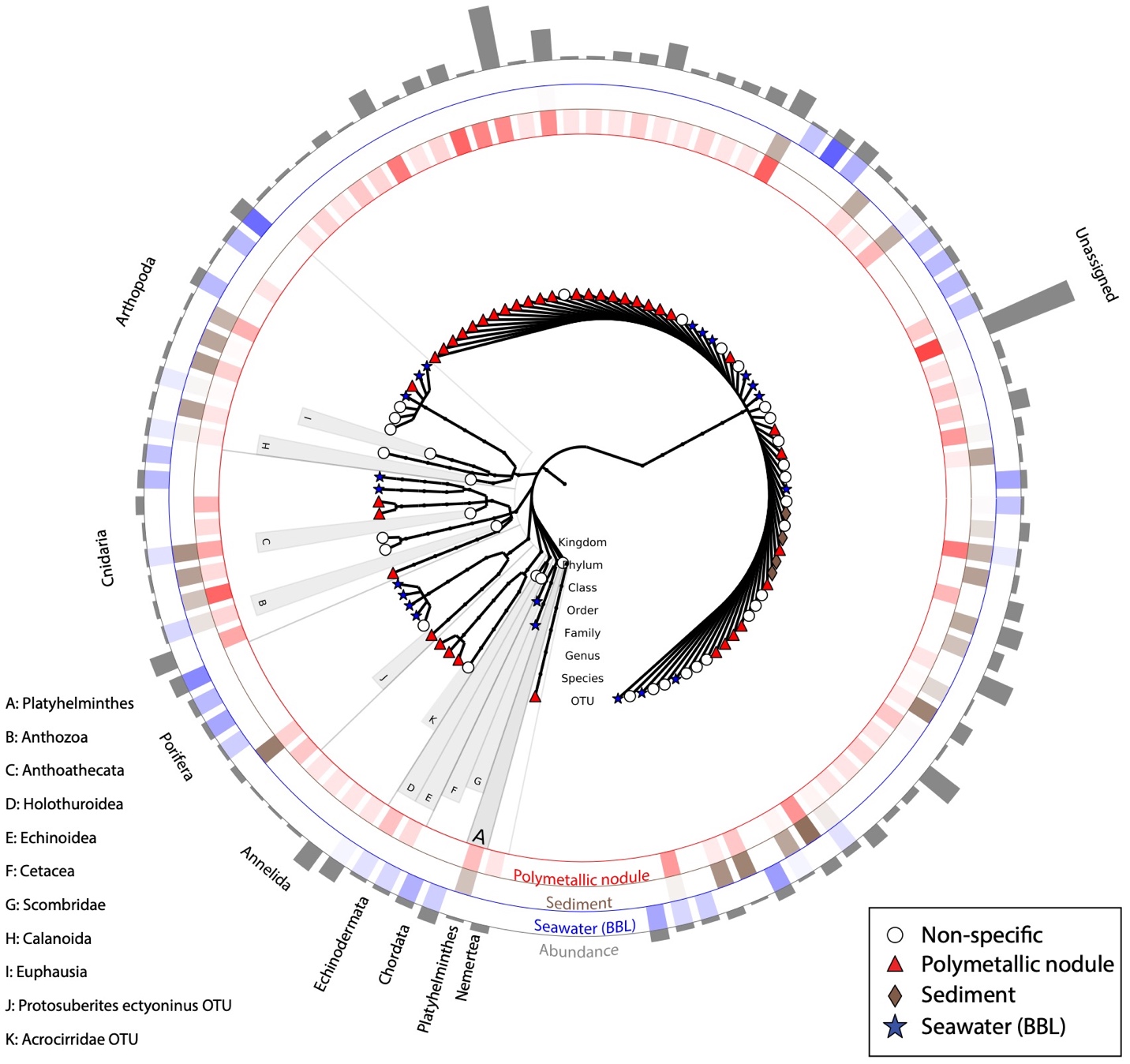


**Figure S3.** Cladogram with circular heatmap and barplots for the metazoan community resolved by COI. The color intensity in the circular heatmap corresponds to mean relative abundance in each sample type across the whole dataset. The bar heights on the outside of the circle are proportional to the mean relative abundance of each taxon within the entire dataset. Taxa found exclusively in one sample type are marked by a corresponding symbol: red triangle for nodules, grey diamond for sediment and blue star for BBL. Those found in more than one sample type are marked by a white circle. The 20 most abundant taxa at the tip of each branch are labelled with letters, and identified to highest taxonomic resolution (key at left). Only taxa found in a minimum of five samples were included. BBL = benthic boundary layer.


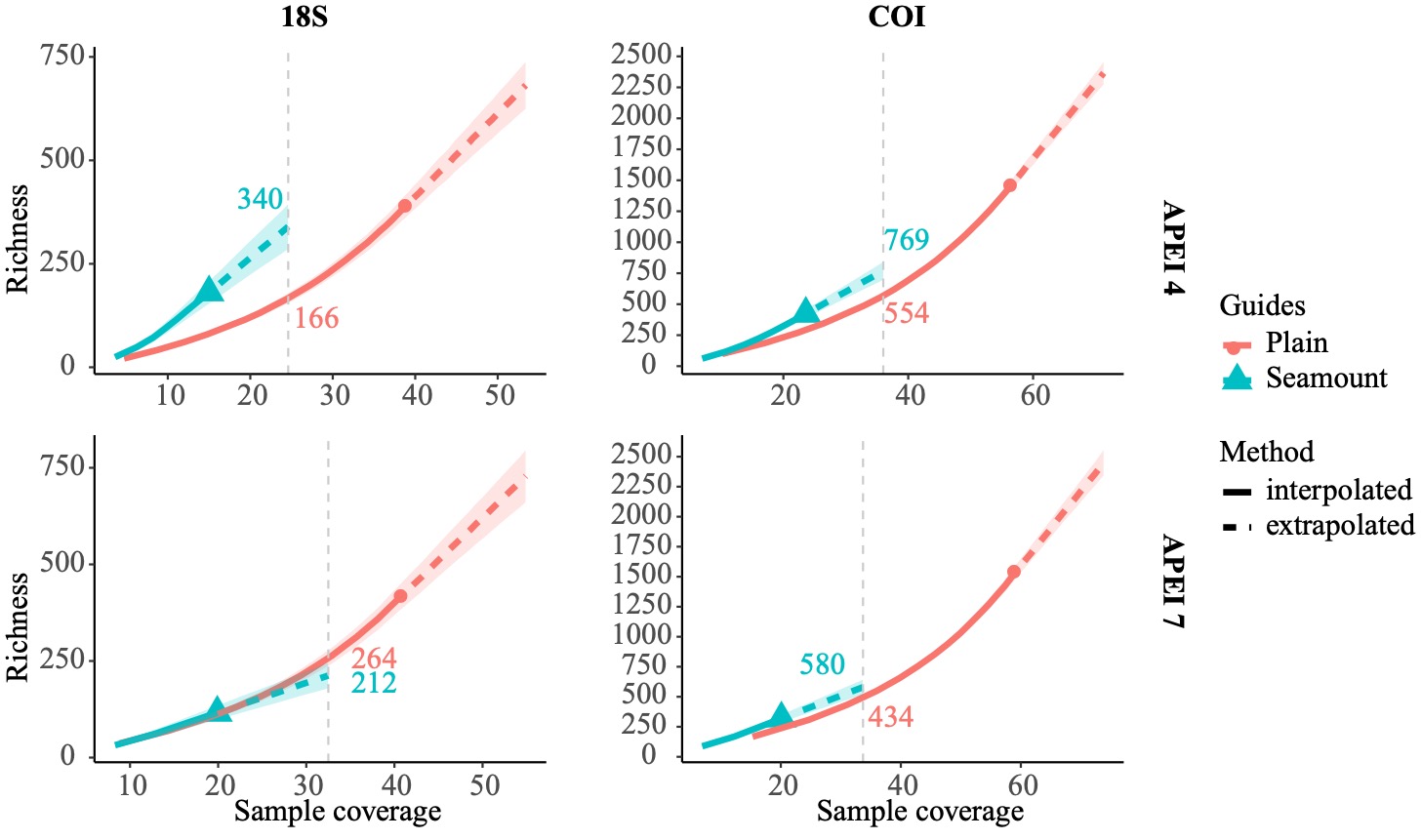


**Figure S4.** Metazoan 18S amplicon sequence variant (ASVs) and COI operational taxonomic unit (COI OTU) gamma diversity of sediment samples per APEI and habitat variable at base sampling coverage. ASV and OTU richness were estimated using Chao2. Shaded colored areas indicate the 95 % confidence intervals obtained using a bootstrap method with 200 replicates. Colored numbers in the plots represent number of ASVs at base coverage.
